# Supplementary material for: Characterization of the Gut Microbiota in Urban Thai Individuals Reveals Enterotype-Specific Signature
Source: Microorganisms. 2023 Jan 5;11(1):136. doi: 10.3390/microorganisms11010136 (PMC9866083; doi:10.3390/microorganisms11010136)
Supplement: Supplementary file 1 [file microorganisms-11-00136-s001.zip › Table S1 - characteristics of lifestyle variables in Thai individuals.pdf]

Table S1: Characteristics of lifestyle variables in Thai individuals

P-values were derived from Fisher's exact test for testing the difference of lifestyle variables between enterotypes.

Variable/Question	Answer	All	EnP	EnB	p-value
N	-	96	32	64	
Gender	Male:Female	29:61	12:17	17:44	0.339
Age	18 ≤ age ≤ 25:25 < age ≤ 35:35 < age ≤ 45:45 < age ≤ 55: > 55 years old<br>(mean ± sd)	20:29:31:6:4 (34.17 ± 10.57)	6:10:10:2:1 (34.31±10.75)	14:19:21:4:3 (34.10±10.56)	0.972
BMI	Underweight (<18.5 kg/m^2):Normal (18.5-24.9 kg/m^2):Overweight (25-29.9 kg/m^2):Obese (>30 kg/m^2)<br>(mean ± sd)	9:48:23:10 (22.58 ± 3.68)	2:16:8:3 (22.78 ± 3.40)	7:32:15:7 (22.48 ± 3.82)	0.884
What is your current dietary pattern?	vegetable and meat:vegetable and meat but not consume raw meat:vegetarian food	62:27:1	22:7:0	40:20:1	0.586
Do you take multi-vitamin?	yes:no	65:23	22:7	43:16	1.000
How often do you have probiotics and prebiotics?	never:rarely (a few times per month):sometimes (1-2 times per week):usually (3-5 times per week):everyday	2:41:27:14:5	0:15:7:7:0	2:26:20:7:5	0.231
What kind of drinking water do you mainly have?	bottled water:tap water	73:10	24:3	49:7	0.933
How often do you exercise?	never:rarely (a few times per month):sometimes (1-2 times per	13:29:30:16:2	5:9:8:6:1	8:20:22:10:1	0.824

	week):usually (3-5 times per week):everyday				
Do you exercise indoor or outdoor?	indoor:outdoor:both:depend on season:never	39:13:23:7:6	13:2:10:1:3	26:11:13:6:3	0.442
How often do you smoke?	never:rarely (a few times per month):sometimes (1-2 times per week):usually (3-5 times per week):everyday	85:2:1:1:0	27:1:0:0:0	58:1:1:1:0	0.786
How often do you drink alcoholic beverage?	never:rarely (a few times per month):sometimes (1-2 times per week):usually (3-5 times per week):everyday	41:42:4:1:0	13:13:2:0:0	28:29:2:1:0	0.819
How often do you use dental floss?	never:rarely (a few times per month):sometimes (1-2 times per week):usually (3-5 times per week):everyday	28:32:8:5:15	10:11:2:2:2	18:21:6:3:13	0.540
How many hours a day do you sleep on average?	less than 5 hours:5-6 hours:6-7 hours:7-8 hours	6:34:32:18	2:15:8:4	4:19:24:14	0.333
How often do you poop?	less than one time per day:once a day:twice a day:three times a day:four times a day	12:55:15:3:3	4:18:3:2:1	8:37:12:1:2	0.549
What does your stool look like?	quite liquid:normal:quite hard:cannot specify	6:66:7	1:20:3	5:46:4	0.432
When was the last time you took antibiotics?	within 1 last week:within 1 last month:2-6 months ago:7 months - 1 year ago:more than 1 year/never	4:10:22:8:13	0:3:6:2:6	4:7:16:6:7	0.624
When was your last influenza vaccination?	within 1 last month:2-6 months ago:7 months - 1 year ago:more than 1 year/never	1:8:6:18	0:2:1:5	1:6:5:13	0.795

Are you taking or have you taken within the last 1 year any hormone regulator? (If used or using, please specify type of hormone regulator)	never:used or using (pills):used or using (drinks)	80:4:1	27:1:0	53:3:1	1.000
In the past 6 months, how much has your body weight changed?	change not more than 2 kg:change 2-5 kg :change more than 5 kg	47:30:12	16:10:3	31:20:9	0.880
Have you ever removed your tonsil?	yes:no	3:85	2:27	1:58	0.252
Have you ever had appendectomy?	yes:no	10:79	3:26	7:53	1.000
Are you currently taking any medication?	yes:no	14:72	5:22	9:50	0.757
Were you born by caesarean section?	yes:no(vaginal birth)	61:18	21:4	40:14	0.497
What was your main food in your infant age (0-6 months)?	breast milk:formulated milk:both	3:10:20	1:5:7	2:5:13	0.655
Are you lactose intolerant?	yes:no	9:74	1:27	8:47	0.261
Are you gluten intolerant?	yes:no	1:82	0:28	1:54	1.000
Do you have any seasonal allergy?	yes:no	22:64	5:24	17:40	0.297

How often do you cook by yourself?	never:rarely (a few times per month):sometimes (1-2 times per week):usually (3-5 times per week):everyday	7:36:21:14:11	2:13:8:4:2	5:23:13:10:9	0.839
How often do you have instant food?	never:rarely (a few times per month):sometimes (1-2 times per week):usually (3-5 times per week):everyday	1:26:18:25:19	0:7:8:8:6	1:19:10:17:13	0.786
How often do you have egg or meat?	never:rarely (a few times per month):sometimes (1-2 times per week):usually (3-5 times per week):everyday	0:0:3:31:56	0:0:3:10:16	0:0:0:21:40	0.060
How often do you have grain?	never:rarely (a few times per month):sometimes (1-2 times per week):usually (3-5 times per week):everyday	6:45:18:14:5	3:14:7:2:2	3:31:11:12:3	0.442
How often do you have vegetable?	never:rarely (a few times per month):sometimes (1-2 times per week):usually (3-5 times per week):everyday	1:13:30:25:20	1:5:12:6:5	0:8:18:19:15	0.367
How often do you have dairy product?	never:rarely (a few times per month):sometimes (1-2 times per week):usually (3-5 times per week):everyday	5:23:26:16:18	2:9:10:4:3	3:14:16:12:15	0.468
How often do you have cow milk substitutes (e.g., soy milk, almond milk, and pistachio milk)?	never:rarely (a few times per month):sometimes (1-2 times per week):usually (3-5 times per week):everyday	15:35:26:4:8	4:12:9:1:3	11:23:17:3:5	0.983

How often do you have sweet?	never:rarely (a few times per month):sometimes (1-2 times per week):usually (3-5 times per week):everyday	0:16:34:23:14	0:4:13:7:4	0:12:21:16:10	0.828
How often do you have red meat?	never:rarely (a few times per month):sometimes (1-2 times per week):usually (3-5 times per week):everyday	9:16:28:22:13	2:9:6:8:4	7:7:22:14:9	0.201
How often do you have poultry?	never:rarely (a few times per month):sometimes (1-2 times per week):usually (3-5 times per week):everyday	1:5:33:42:6	1:0:15:10:3	0:5:18:32:3	**0.040***
How often do you have seafood?	never:rarely (a few times per month):sometimes (1-2 times per week):usually (3-5 times per week):everyday	1:31:38:15:1	0:14:10:4:0	1:17:28:11:1	0.421
How often do you have salty snack?	never:rarely (a few times per month):sometimes (1-2 times per week):usually (3-5 times per week):everyday	0:40:34:11:2	0:14:10:4:1	0:26:24:7:1	0.851
How often do you have high sugar containing food?	never:rarely (a few times per month):sometimes (1-2 times per week):usually (3-5 times per week):everyday	0:41:33:10:3	0:18:7:3:0	0:23:26:7:3	0.135
Which part of egg do you eat?	whole:partial	84:3	27:1	57:2	0.763
How often do you drink soft drink?	never:rarely (a few times per month):sometimes (1-2 times per	56:31:3:0:0	18:11:0:0:0	38:20:3:0:0	0.649

	week):usually (3-5 times per week):everyday				
How often do you have sweetener?	never:rarely (a few times per month):sometimes (1-2 times per week):usually (3-5 times per week):everyday	25:30:19:7:5	9:8:8:1:3	16:22:11:6:2	0.426
How often do you drink 1 liter of water a day?	never:rarely (a few times per month):sometimes (1-2 times per week):usually (3-5 times per week):everyday	1:10:19:19:39	1:5:7:5:11	0:5:12:14:28	0.388
