# Supplementary material for: Characterization of the Gut Microbiota in Urban Thai Individuals Reveals Enterotype-Specific Signature
Source: Microorganisms. 2023 Jan 5;11(1):136. doi: 10.3390/microorganisms11010136 (PMC9866083; doi:10.3390/microorganisms11010136)
Supplement: Supplementary file 1 [file microorganisms-11-00136-s001.zip › Table S2 - the number of reads remaining in each step of microbiome analysis.pdf]

Table S2: The number of reads remaining in each step of microbiome analysis

Parameters	Raw	Filtered	Denoised	Merged	Non-chimeric
Minimum	37,029	26,814	26,611	26,388	25,299
1st Quartile	59,533.75	44,776	44,488.5	43,248	35,618.5
Median	68,379	51,598.5	50,896	48,359	40,109
Mean	68,866.76	52,095.02	51,483.85	49,348.84	41,445.70
3rd Quartile	75,320	57,094	56,231.25	54,329.75	44,936.25
Maximum	120,475	95,353	94,667	92,983	86,823
Total	6,611,209	5,001,122	4,942,450	4,737,489	3,978,787
