# Supplementary material for: Characterization of the Gut Microbiota in Urban Thai Individuals Reveals Enterotype-Specific Signature
Source: Microorganisms. 2023 Jan 5;11(1):136. doi: 10.3390/microorganisms11010136 (PMC9866083; doi:10.3390/microorganisms11010136)
Supplement: Supplementary file 1 [file microorganisms-11-00136-s001.zip › Table S3 - amplicon sequence length distribution.pdf]

**Table S3: Amplicon sequence length distribution**

| Percentile | Sequence length after preprocessing |
|---|---|
| 2% | 252 |
| 9% | 253 |
| 25% | 253 |
| 50% | 253 |
| 75% | 253 |
| 91% | 253 |
| 98% | 254 |
