# Supplementary material for: Characterization of the Gut Microbiota in Urban Thai Individuals Reveals Enterotype-Specific Signature
Source: Microorganisms. 2023 Jan 5;11(1):136. doi: 10.3390/microorganisms11010136 (PMC9866083; doi:10.3390/microorganisms11010136)
Supplement: Supplementary file 1 [file microorganisms-11-00136-s001.zip › Table S5 - Fisherí»s exact test of bacteria at least 1% abundance with age group, BMI level, gender, and enterotype.pdf]

Table S5: Fisher's exact test of bacteria at least 1% abundance with age group, BMI level, gender, and enterotype

The numbers shown in the table are p-values of Fisher's exact test between taxa and lifestyle variables. The asterisk (*) indicates the significant p-value.

Taxa	Enterotype	Age group	Gender	BMI level
[Eubacterium] coprostanoligenes group	1.000	0.485	0.206	0.934
[Eubacterium] eligens group	0.005*	0.932	0.062	0.453
[Eubacterium] ruminantium group	0.683	0.383	0.660	0.714
[Eubacterium] xylanophilum group	1.000	0.333	1.000	1.000
[Ruminococcus] gnavus group	1.000	0.656	1.000	0.100
[Ruminococcus] torques group	0.551	1.000	1.000	0.718
Acidaminococcus	0.326	0.647	0.745	0.753
Akkermansia	0.713	0.512	1.000	0.469
Alistipes	0.003*	0.540	0.358	0.629
Alloprevotella	0.002*	0.083	0.106	0.791
Anaerosporobacter	1.000	1.000	1.000	1.000
Anaerostipes	1.000	0.333	1.000	1.000
Anaerovibrio	0.109	0.621	0.101	0.443
Azospirillum sp. 47_25	1.000	1.000	1.000	1.000
Bacteroides	0.003*	0.327	0.323	0.898
Barnesiella	0.049*	0.397	0.429	0.380
Bifidobacterium	0.093	0.143	0.492	0.300
Bilophila	0.548	0.732	1.000	0.252
Blautia	0.262	0.643	0.709	0.627
Butyricicoccus	1.000	0.111	0.322	0.467
Butyricimonas	0.551	0.776	0.543	0.443
Christensenellaceae R-7 group	0.373	0.320	0.763	0.172
Collinsella	0.551	1.000	1.000	0.323
Coprococcus 2	0.551	0.776	1.000	0.152
Desulfovibrio	0.660	0.434	0.660	0.779
Dialister	0.527	0.682	1.000	0.418

Dorea	0.333	0.656	0.322	1.000
Elusimicrobium	0.333	0.333	0.322	1.000
Enterobacteriaceae	0.487	0.722	0.468	0.950
Escherichia-Shigella	0.230	0.897	1.000	0.699
Faecalibacterium	0.715	0.213	0.266	0.021*
Flavonifractor	0.551	0.776	0.543	0.718
Fusicatenibacter	0.548	0.732	0.548	0.779
Fusobacterium	0.056	0.263	0.513	0.444
Gastranaerophilales	0.109	1.000	0.543	0.718
Haemophilus	0.298	0.847	0.548	0.090
Hungatella	1.000	0.656	1.000	1.000
Lachnoclostridium	0.017*	0.358	0.763	0.878
Lachnospira	0.011*	0.637	0.059	0.182
Lachnospiraceae NK4A136 group	0.059	0.091	0.451	0.423
Lachnospiraceae UCG-001	1.000	0.333	1.000	1.000
Lachnospiraceae UCG-003	0.333	1.000	0.322	0.467
Lachnospiraceae UCG-004	0.551	0.476	0.543	0.020*
Lachnospiraceae UCG-006	0.551	0.111	0.322	0.467
Lachnospiraceae UCG-010	0.548	0.732	1.000	0.109
Lachnospiraceae	2.45E-05*	0.708	0.793	0.565
Megamonas	1.000	0.139	0.349	0.520
Megasphaera	0.498	0.959	0.492	0.182
Mitsuokella	0.551	0.165	0.543	0.718
Muribaculaceae gut metagenome	1.000	0.111	0.322	0.467
Muribaculaceae metagenome	0.662	0.796	0.323	0.708
Negativibacillus	1.000	1.000	0.322	1.000
Oscillibacter	1.000	1.000	1.000	0.467
Parabacteroides	0.001*	0.204	0.015*	0.447
Paraprevotella	0.017*	0.772	0.766	0.840
Parasutterella	0.713	0.578	0.709	0.706

Phascolarctobacterium	0.011*	0.744	0.632	0.686
Prevotella 2	6.90E-04*	0.420	0.166	0.162
Prevotella 7	1.000	0.656	1.000	1.000
Prevotella 9	2.59E-18*	0.965	1.000	0.835
Prevotellaceae NK3B31 group	1.000	1.000	1.000	0.133
Prevotellaceae UCG-001	0.551	0.360	1.000	1.000
Rhodospirillales gut metagenome	0.548	5.11E-04*	0.548	0.339
Romboutsia	1.000	0.333	0.322	1.000
Roseburia	0.003*	0.610	1.000	0.763
Ruminiclostridium 6	0.548	0.621	1.000	1.000
Ruminococcaceae NK4A214 group	1.000	0.448	0.097	0.844
Ruminococcaceae UCG-002	0.518	0.943	1.000	0.437
Ruminococcaceae UCG-003	0.174	0.789	0.660	0.481
Ruminococcaceae UCG-005	0.208	0.616	0.537	1.000
Ruminococcaceae UCG-010	0.333	1.000	0.322	0.467
Ruminococcaceae UCG-014	1.000	0.625	1.000	0.632
Ruminococcus 1	0.327	0.520	1.000	0.450
Ruminococcus 2	0.049*	0.458	0.429	0.706
Streptococcus	1.000	1.000	1.000	0.211
Subdoligranulum	0.047*	0.080	0.250	0.197
Succinivibrio	0.106	0.894	0.592	0.733
Sutterella	5.96E-05*	0.873	0.651	0.401
Tyzzerella	0.551	1.000	0.543	0.718
uncultured Lachnospiraceae	0.551	0.776	1.000	0.718
uncultured Muribaculaceae bacterium	0.548	0.165	0.101	0.718
uncultured Porphyromonadaceae bacterium	1.000	1.000	0.543	0.152
uncultured Prevotellaceae	0.329	1.000	0.656	0.627
uncultured Puniceicoccaceae	1.000	1.000	1.000	0.467
uncultured Ruminococcaceae	0.548	1.000	0.548	0.339

Veillonella	0.551	0.476	1.000	0.020*
Victivallis	1.000	1.000	1.000	1.000
Number of significant taxa (p-value < 0.05)	17	1	1	3
