# Supplementary material for: Characterization of the Gut Microbiota in Urban Thai Individuals Reveals Enterotype-Specific Signature
Source: Microorganisms. 2023 Jan 5;11(1):136. doi: 10.3390/microorganisms11010136 (PMC9866083; doi:10.3390/microorganisms11010136)
Supplement: Supplementary file 1 [file microorganisms-11-00136-s001.zip › Table S6 - association between microbial taxa and diversity indices using Spearmaní»s correlation coefficient.pdf]

Table S6: Association between microbial taxa present 1% relative abundance at least one sample, and diversity indices using Spearman's correlation coefficient.

Values in the table: Correlation (P-value)

Taxa	Shannon	Simpson	Chao1	Pielou
[Eubacterium] coprostanoligenes group	0.572 (1.19E-09)*	0.47 (1.39E-06)*	0.791 (9.98E-22)*	0.498 (2.40E-07)*
[Eubacterium] eligens group	0.269 (0.008)*	0.221 (0.03)*	0.235 (0.021)*	0.256 (0.012)*
[Eubacterium] ruminantium group	0.273 (0.007)*	0.219 (0.032)*	0.405 (4.19E-05)*	0.224 (0.028)*
[Eubacterium] xylanophilum group	0.497 (2.67E-07)*	0.433 (1.04E-05)*	0.502 (1.85E-07)*	0.472 (1.22E-06)*
[Ruminococcus] gnavus group	-0.125 (0.224)	-0.117 (0.257)	-0.236 (0.02)*	-0.086 (0.406)
[Ruminococcus] torques group	0.498 (2.41E-07)*	0.453 (3.63E-06)*	0.534 (2.17E-08)*	0.472 (1.22E-06)*
Acidaminococcus	0.031 (0.763)	0.039 (0.708)	0.146 (0.155)	0.002 (0.982)
Akkermansia	0.336 (8.27E-04)*	0.276 (0.006)*	0.376 (1.57E-04)*	0.322 (0.001)*
Alistipes	0.601 (9.95E-11)*	0.496 (2.73E-07)*	0.596 (1.43E-10)*	0.568 (1.62E-09)*
Alloprevotella	0.017 (0.866)	-0.018 (0.86)	0.166 (0.105)	-0.017 (0.871)
Anaerosporobacter	0.251 (0.014)*	0.27 (0.008)*	0.255 (0.012)*	0.237 (0.02)*
Anaerostipes	0.256 (0.012)*	0.243 (0.017)*	0.146 (0.155)	0.254 (0.013)*
Anaerovibrio	0.066 (0.526)	0.043 (0.681)	0.108 (0.295)	0.042 (0.684)
Azospirillum sp. 47_25	0.163 (0.112)	0.154 (0.133)	0.112 (0.278)	0.154 (0.133)
Bacteroides	0.039 (0.707)	0.023 (0.824)	-0.234 (0.022)*	0.098 (0.34)
Barnesiella	0.424 (1.69E-05)*	0.364 (2.67E-04)*	0.442 (6.65E-06)*	0.412 (3.07E-05)*
Bifidobacterium	0.471 (1.27E-06)*	0.42 (2.07E-05)*	0.515 (7.98E-08)*	0.42 (2.01E-05)*
Bilophila	0.341 (6.65E-04)*	0.269 (0.008)*	0.246 (0.016)*	0.342 (6.43E-04)*
Blautia	0.489 (4.41E-07)*	0.422 (1.86E-05)*	0.458 (2.78E-06)*	0.461 (2.24E-06)*
Butyricicoccus	0.318 (0.002)*	0.258 (0.011)*	0.199 (0.052)	0.34 (7.08E-04)*
Butyricimonas	0.477 (8.75E-07)*	0.388 (9.45E-05)*	0.533 (2.32E-08)*	0.441 (6.79E-06)*
Christensenellaceae R-7 group	0.631 (5.62E-12)*	0.529 (2.91E-08)*	0.808 (2.78E-23)*	0.553 (5.34E-09)*
Collinsella	0.482 (6.67E-07)*	0.474 (1.05E-06)*	0.395 (6.91E-05)*	0.478 (8.46E-07)*
Coprococcus 2	0.373 (1.86E-04)*	0.319 (0.002)*	0.519 (5.93E-08)*	0.337 (7.92E-04)*

Taxa	Shannon	Simpson	Chao1	Pielou
Desulfovibrio	0.365 (2.58E-04)*	0.277 (0.006)*	0.427 (1.43E-05)*	0.333 (9.23E-04)*
Dialister	0.198 (0.053)	0.126 (0.222)	0.373 (1.84E-04)*	0.152 (0.14)
Dorea	0.522 (4.82E-08)*	0.456 (3.10E-06)*	0.589 (2.86E-10)*	0.478 (8.38E-07)*
Elusimicrobium	-0.033 (0.751)	-0.064 (0.537)	0.027 (0.795)	-0.054 (0.604)
Enterobacteriaceae	0.083 (0.424)	0.092 (0.371)	-0.003 (0.98)	0.077 (0.453)
Escherichia-Shigella	0.269 (0.008)*	0.253 (0.013)*	0.237 (0.02)*	0.271 (0.008)*
Faecalibacterium	0.274 (0.007)*	0.345 (5.80E-04)*	0.123 (0.231)	0.279 (0.006)*
Flavonifractor	0.15 (0.146)	0.116 (0.26)	-0.033 (0.752)	0.182 (0.077)
Fusicatenibacter	0.598 (1.23E-10)*	0.56 (2.89E-09)*	0.547 (8.19E-09)*	0.58 (5.86E-10)*
Fusobacterium	-0.306 (0.002)*	-0.226 (0.027)*	-0.493 (3.40E-07)*	-0.237 (0.02)*
Gastranaerophilales	0.127 (0.218)	0.096 (0.352)	0.218 (0.033)*	0.088 (0.395)
Haemophilus	-0.085 (0.411)	-0.019 (0.855)	-0.108 (0.293)	-0.096 (0.354)
Hungatella	0.029 (0.776)	0.037 (0.721)	-0.037 (0.722)	0.049 (0.636)
Lachnoclostridium	0.287 (0.005)*	0.264 (0.009)*	-0.019 (0.856)	0.336 (8.08E-04)*
Lachnospira	0.347 (5.39E-04)*	0.273 (0.007)*	0.285 (0.005)*	0.328 (0.001)*
Lachnospiraceae NK4A136 group	0.47 (1.39E-06)*	0.452 (3.84E-06)*	0.46 (2.47E-06)*	0.442 (6.43E-06)*
Lachnospiraceae UCG-001	0.463 (2.01E-06)*	0.413 (2.94E-05)*	0.49 (4.13E-07)*	0.442 (6.40E-06)*
Lachnospiraceae UCG-003	0.139 (0.176)	0.127 (0.219)	0.173 (0.092)	0.123 (0.233)
Lachnospiraceae UCG-004	0.198 (0.053)	0.179 (0.08)	0.168 (0.102)	0.192 (0.061)
Lachnospiraceae UCG-006	0.343 (6.34E-04)*	0.348 (5.18E-04)*	0.194 (0.059)	0.364 (2.61E-04)*
Lachnospiraceae UCG-010	0.507 (1.32E-07)*	0.508 (1.30E-07)*	0.486 (5.13E-07)*	0.497 (2.66E-07)*
Lachnospiraceae	0.462 (2.13E-06)*	0.446 (5.22E-06)*	0.192 (0.06)	0.511 (1.01E-07)*
Megamonas	0.04 (0.7)	0.03 (0.775)	0.02 (0.85)	0.057 (0.579)
Megasphaera	-0.079 (0.446)	-0.102 (0.322)	0.121 (0.241)	-0.121 (0.242)
Mitsuokella	0.15 (0.145)	0.138 (0.18)	0.171 (0.096)	0.136 (0.186)
Muribaculaceae gut metagenome	0.194 (0.058)	0.136 (0.187)	0.241 (0.018)*	0.18 (0.079)
Muribaculaceae metagenome	-0.094 (0.36)*	-0.087 (0.401)	-0.012 (0.91)	-0.107 (0.299)
Negativibacillus	0.412 (3.07E-05)*	0.325 (0.001)*	0.582 (5.19E-10)*	0.363 (2.74E-04)*
Oscillibacter	0.584 (4.34E-10)*	0.488 (4.48E-07)*	0.587 (3.28E-10)*	0.547 (7.81E-09)*

Taxa	Shannon	Simpson	Chao1	Pielou
Parabacteroides	0.402 (5.02E-05)*	0.345 (5.78E-04)*	0.274 (0.007)*	0.422 (1.82E-05)*
Paraprevotella	0.203 (0.047)*	0.129 (0.21)	0.302 (0.003)*	0.18 (0.08)
Parasutterella	0.106 (0.302)	0.07 (0.497)	0.084 (0.417)	0.098 (0.343)
Phascolarctobacterium	0.225 (0.028)*	0.24 (0.019)*	0.068 (0.508)	0.243 (0.017)*
Prevotella 2	0.167 (0.103)	0.166 (0.107)	0.211 (0.039)*	0.122 (0.236)
Prevotella 7	0 (0.998)	-0.003 (0.975)	0.047 (0.653)	-0.029 (0.776)
Prevotella 9	-0.316 (0.002)	-0.336 (8.20E-04)*	0.022 (0.829)	-0.373 (1.85E-04)*
Prevotellaceae NK3B31 group	0.044 (0.672)	0.038 (0.716)	0.121 (0.242)	0.031 (0.766)
Prevotellaceae UCG-001	0.111 (0.281)	0.102 (0.322)	0.15 (0.145)	0.098 (0.342)
Rhodospirillales gut metagenome	0.142 (0.169)	0.134 (0.191)	0.128 (0.215)	0.132 (0.199)
Romboutsia	0.203 (0.047)*	0.234 (0.022)*	0.21 (0.04)*	0.178 (0.083)
Roseburia	0.376 (1.61E-04)*	0.344 (6.07E-04)*	0.317 (0.002)*	0.359 (3.24E-04)*
Ruminiclostridium 6	0.44 (7.24E-06)*	0.397 (6.14E-05)*	0.483 (6.20E-07)*	0.404 (4.42E-05)*
Ruminococcaceae NK4A214 group	0.621 (1.45E-11)*	0.523 (4.68E-08)*	0.788 (1.80E-21)*	0.558 (3.52E-09)*
Ruminococcaceae UCG-002	0.642 (1.79E-12)*	0.567 (1.68E-09)*	0.798 (2.30E-22)*	0.58 (6.09E-10)*
Ruminococcaceae UCG-003	0.609 (4.43E-11)*	0.565 (1.96E-09)*	0.62 (1.58E-11)*	0.568 (1.61E-09)*
Ruminococcaceae UCG-005	0.664 (1.59E-13)*	0.546 (8.74E-09)*	0.804 (5.69E-23)*	0.6 (1.03E-10)*
Ruminococcaceae UCG-010	0.537 (1.67E-08)*	0.454 (3.31E-06)*	0.704 (1.27E-15)*	0.473 (1.12E-06)*
Ruminococcaceae UCG-014	0.42 (2.05E-05)*	0.377 (1.51E-04)*	0.453 (3.65E-06)*	0.39 (8.52E-05)*
Ruminococcus 1	0.578 (6.73E-10)*	0.499 (2.23E-07)*	0.676 (3.91E-14)*	0.521 (5.30E-08)*
Ruminococcus 2	0.472 (1.17E-06)*	0.43 (1.23E-05)*	0.324 (0.001)*	0.471 (1.28E-06)*
Streptococcus	0.244 (0.016)*	0.229 (0.025)	0.232 (0.023)*	0.221 (0.03)*
Subdoligranulum	0.586 (3.64E-10)*	0.522 (4.80E-08)*	0.561 (2.72E-09)*	0.545 (9.37E-09)*
Succinivibrio	0.045 (0.665)	0.066 (0.523)	0.091 (0.377)	0.023 (0.822)
Sutterella	0.302 (0.003)*	0.247 (0.015)*	0.222 (0.03)*	0.302 (0.003)*
Tyzzerella	-0.042 (0.685)	-0.032 (0.754)	-0.21 (0.04)*	0.013 (0.904)
uncultured Lachnospiraceae	0.609 (4.48E-11)*	0.546 (8.82E-09)*	0.538 (1.63E-08)*	0.602 (8.79E-11)*
uncultured Muribaculaceae bacterium	0.268 (0.008)*	0.208 (0.042)*	0.292 (0.004)*	0.247 (0.015)*

**Taxa**	**Shannon**	**Simpson**	**Chao1**	**Pielou**
uncultured Porphyromonadaceae bacterium	0.095 (0.359)	0.116 (0.26)	0.073 (0.478)	0.094 (0.36)
uncultured Prevotellaceae	0.142 (0.168)	0.134 (0.193)	0.17 (0.098)	0.118 (0.254)
uncultured Puniceicoccaceae	0.314 (0.002)*	0.271 (0.008)*	0.38 (1.34E-04)*	0.297 (0.003)*
uncultured Ruminococcaceae	0.446 (5.36E-06)*	0.358 (3.44E-04)*	0.603 (7.98E-11)*	0.393 (7.56E-05)*
Veillonella	0.023 (0.824)	0.067 (0.517)	-0.12 (0.243)	0.056 (0.585)
Victivallis	0.422 (1.88E-05)*	0.377 (1.52E-04)*	0.549 (7.17E-09)*	0.377 (1.53E-04)*
Number of significant taxa which have correlation in criteria ($\|\rho\| \geq 0.35$ and p-value < 0.05)	35	30	36	35
Number of significant taxa which have positive correlation ($\rho \geq 0.35$ and p-value < 0.05)	35	30	35	34
Number of significant taxa which have negative correlation ($\rho \leq -0.35$ and p-value < 0.05)	0	0	1	1
