# Supplementary material for: Characterization of the Gut Microbiota in Urban Thai Individuals Reveals Enterotype-Specific Signature
Source: Microorganisms. 2023 Jan 5;11(1):136. doi: 10.3390/microorganisms11010136 (PMC9866083; doi:10.3390/microorganisms11010136)
Supplement: Supplementary file 1 [file microorganisms-11-00136-s001.zip › Table S7 - cluster number of belonging taxa.pdf]

Table S7: Cluster number of belonging taxa

Taxa	EnP	EnB	Overall
D_5__Bifidobacterium	1	1	1
D_5__Collinsella	1	2	1
D_5__Bacteroides	2	3	2
D_5__Barnesiella	2	4	1
D_5__Butyricimonas	2	4	1
D_5__Muribaculaceae.gut.metagenome.	2	4	1
D_5__Muribaculaceae.metagenome.	3	2	3
D_5__uncultured.Porphyromonadaceae.bacterium	4	5	1
D_5__uncultured.Muribaculaceae.bacterium.	2	4	1
D_5__Alloprevotella	4	2	3
D_5__Paraprevotella	2	4	1
D_5__Prevotella.2	4	1	3
D_5__Prevotella.7	4	1	3
D_5__Prevotella.9	3	1	3
D_5__Prevotellaceae.NK3B31.group	4	5	3
D_5__Prevotellaceae.UCG.001	1	2	1
D_5__uncultured.Prevotellaceae.	4	2	3
D_5__Alistipes	2	1	1
D_5__Parabacteroides	2	1	1
D_3__Gastranaerophilales.	4	6	3
D_5__Elusimicrobium	4	1	3
D_5__Streptococcus	3	1	1
D_5__Christensenellaceae.R.7.group	1	4	1
D_5__Anaerosporobacter	1	4	1
D_5__Anaerostipes	2	6	1
D_5__Blautia	1	6	1
D_5__Coprococcus.2	4	4	1

D_5__Dorea	1	4	1
D_5__Fusicatenibacter	2	4	1
D_5__Hungatella	3	3	2
D_5__Lachnoclostridium	2	6	1
D_5__Lachnospira	2	4	1
D_5__Lachnospiraceae.NK4A136.group	1	6	1
D_5__Lachnospiraceae.UCG.001	4	4	1
D_5__Lachnospiraceae.UCG.003	1	4	1
D_5__Lachnospiraceae.UCG.004	1	6	1
D_5__Lachnospiraceae.UCG.006	2	6	1
D_5__Lachnospiraceae.UCG.010	2	4	1
D_5__Roseburia	1	6	1
D_5__Tyzzerella	3	3	2
D_5__.Eubacterium..eligens.group	1	2	1
D_5__.Eubacterium..ruminantium.group	1	4	1
D_5__.Eubacterium..xylanophilum.group	1	4	1
D_5__.Ruminococcus..gnavus.group	3	3	2
D_5__.Ruminococcus..torques.group	1	4	1
D_5__uncultured.Lachnospiraceae.	1	2	1
D_4__Lachnospiraceae.	2	6	1
D_5__Romboutsia	1	5	1
D_5__Butyricicoccus	2	6	1
D_5__Faecalibacterium	2	6	1
D_5__Flavonifractor	2	3	2
D_5__Negativibacillus	3	1	1
D_5__Oscillibacter	1	1	1
D_5__Ruminiclostridium.6	1	4	1
D_5__Ruminococcaceae.NK4A214.group	1	4	1
D_5__Ruminococcaceae.UCG.002	1	4	1

D_5__Ruminococcaceae.UCG.003	2	4	1
D_5__Ruminococcaceae.UCG.005	1	4	1
D_5__Ruminococcaceae.UCG.010	1	4	1
D_5__Ruminococcaceae.UCG.014	4	4	1
D_5__Ruminococcus.1	1	4	1
D_5__Ruminococcus.2	4	6	1
D_5__Subdoligranulum	2	4	1
D_5__.Eubacterium..coprostanoligenes.group	1	4	1
D_5__uncultured.Ruminococcaceae.	4	4	1
D_5__Acidaminococcus	3	1	3
D_5__Phascolarctobacterium	4	2	1
D_5__Anaerovibrio	4	1	3
D_5__Dialister	3	1	3
D_5__Megamonas	3	2	3
D_5__Megasphaera	3	1	3
D_5__Mitsuokella	4	4	3
D_5__Veillonella	3	5	1
D_5__Fusobacterium	3	5	2
D_5__Victivallis	4	4	1
D_5__Azospirillum.sp..47_25	1	2	1
D_5__Rhodospirillales.gut.metagenome.	1	3	1
D_5__Bilophila	1	3	1
D_5__Desulfovibrio	1	4	1
D_5__Succinivibrio	4	1	3
D_5__Parasutterella	2	3	1
D_5__Sutterella	4	2	1
D_5__Escherichia.Shigella	2	5	1
D_4__Enterobacteriaceae.	4	5	1
D_5__Haemophilus	3	5	1

D_5__uncultured.Puniceicoccaceae.	4	4	1
D_5__Akkermansia	3	4	1
