# Supplementary material for: Characterization of the Gut Microbiota in Urban Thai Individuals Reveals Enterotype-Specific Signature
Source: Microorganisms. 2023 Jan 5;11(1):136. doi: 10.3390/microorganisms11010136 (PMC9866083; doi:10.3390/microorganisms11010136)
Supplement: Supplementary file 1 [file microorganisms-11-00136-s001.zip › Table S8 - number of taxa (nodes), interactions (edges), and average correlation in individual clusters.pdf]

Table S8: Number of taxa (nodes), interactions (edges), and average correlation in individual clusters

Cluster	EnP			EnB		
	Nodes	Edges	Average correlation	Nodes	Edges	Average correlation
1st	24	68	0.6168	7	2	0.5551
2nd	16	25	0.5879	1	0	0
3rd	8	2	0.5416	2	0	0
4th	19	18	0.5904	25	78	0.5790
5th	-	-	-	4	2	0.5382
6th	-	-	-	3	1	0.5107
