# Supplementary figures and images for: Characterization of the Gut Microbiota in Urban Thai Individuals Reveals Enterotype-Specific Signature
Source: Microorganisms. 2023 Jan 5;11(1):136. doi: 10.3390/microorganisms11010136 (PMC9866083; doi:10.3390/microorganisms11010136)

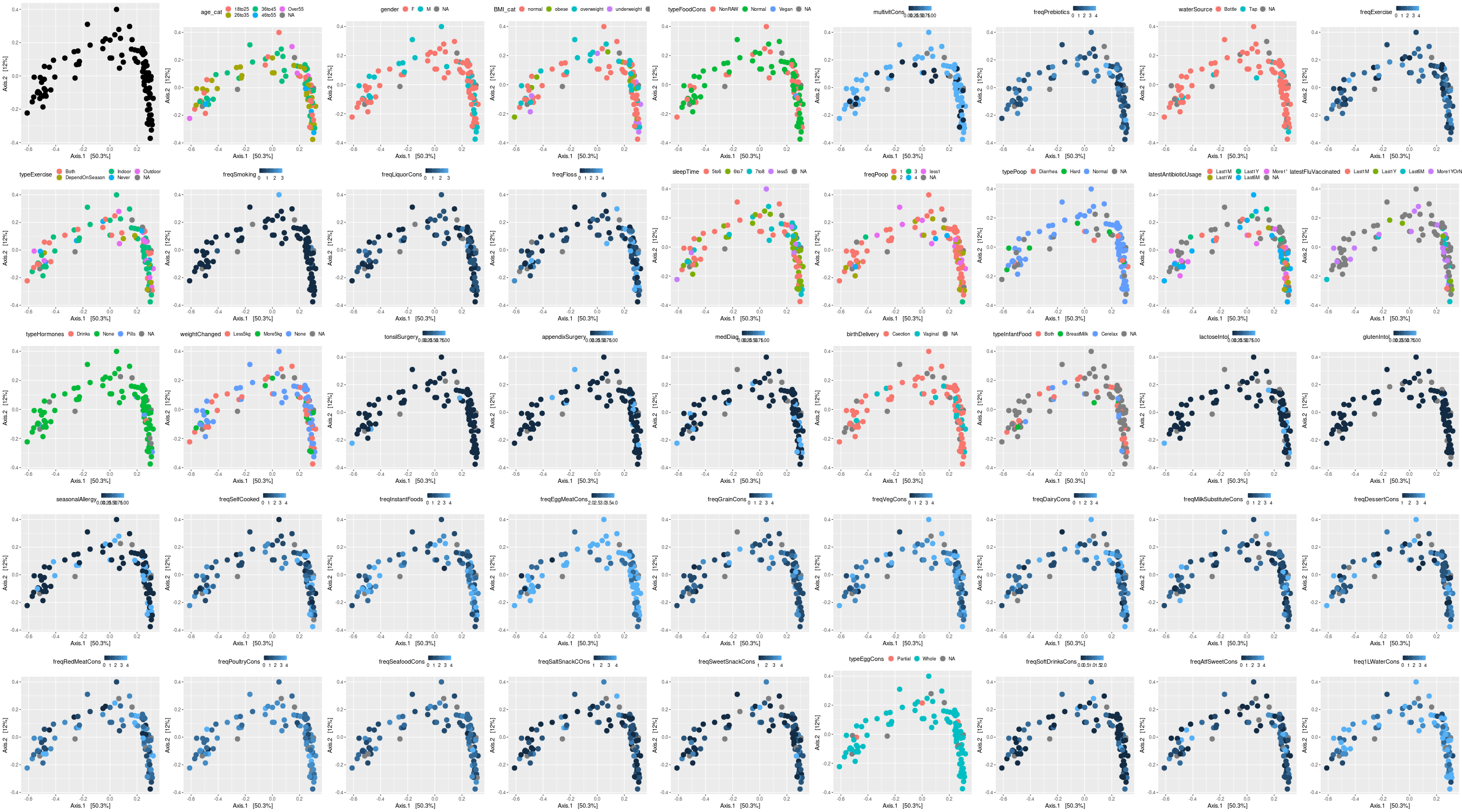

Supplement: Supplementary file 1 [file microorganisms-11-00136-s001.zip › Figure S1 - the association of gut microbiota and host lifestyle parameters using PCoA visualization.png]

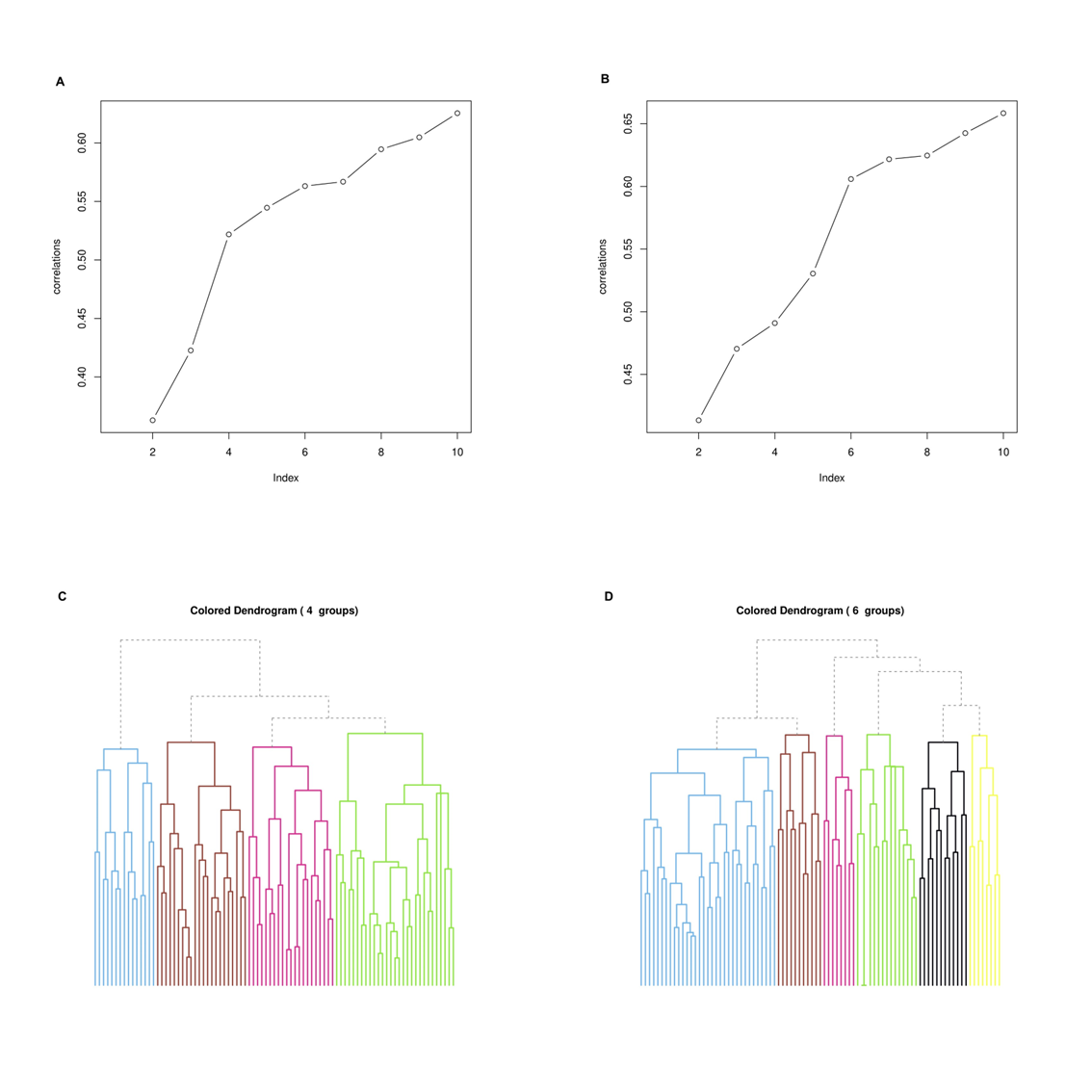

Supplement: Supplementary file 1 [file microorganisms-11-00136-s001.zip › Figure S2 - the clustering of gut microbiome profiles using hierarchical clustering analysis.PNG]

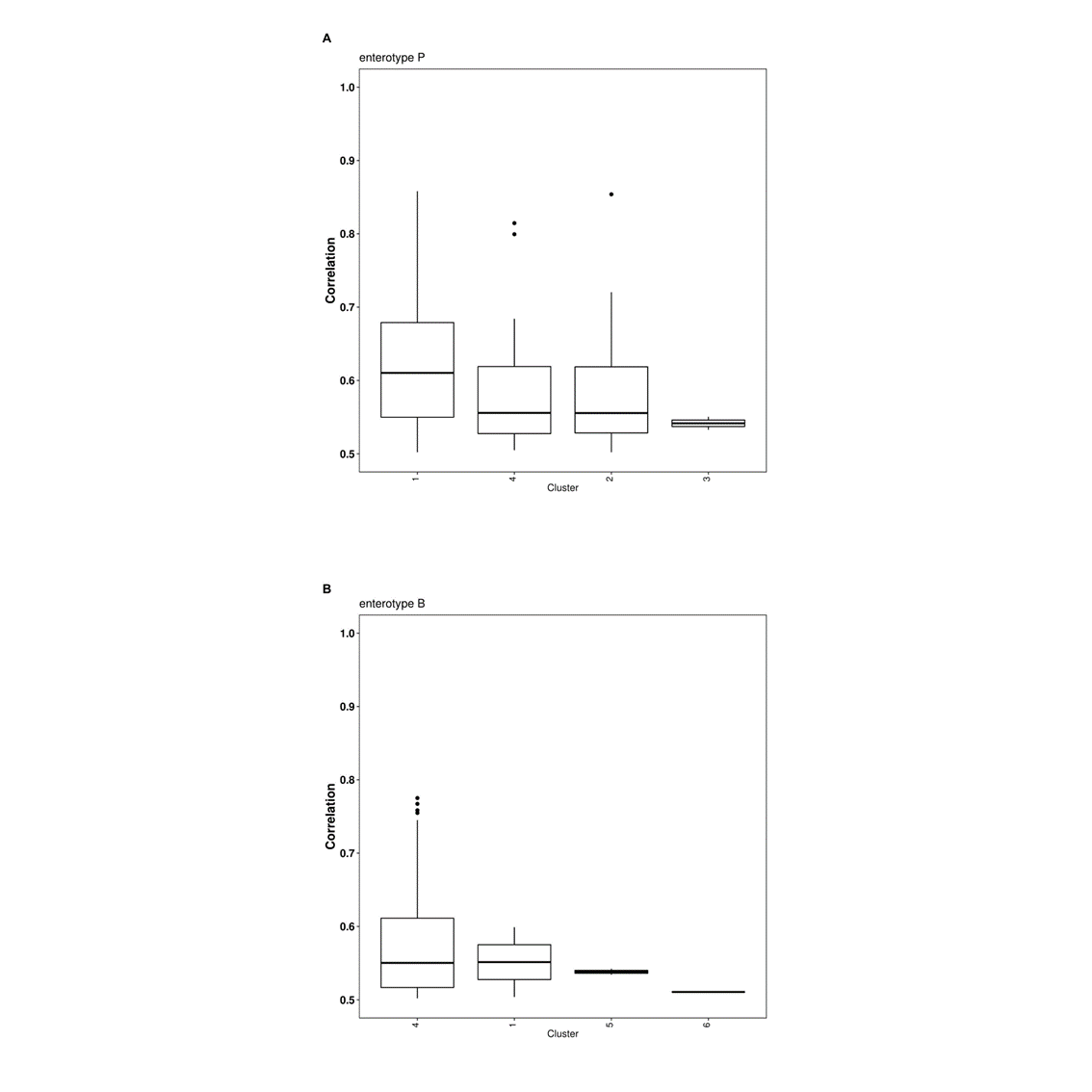

Supplement: Supplementary file 1 [file microorganisms-11-00136-s001.zip › Figure S3 - identification of the highest correlated cluster in enterotypes.PNG]

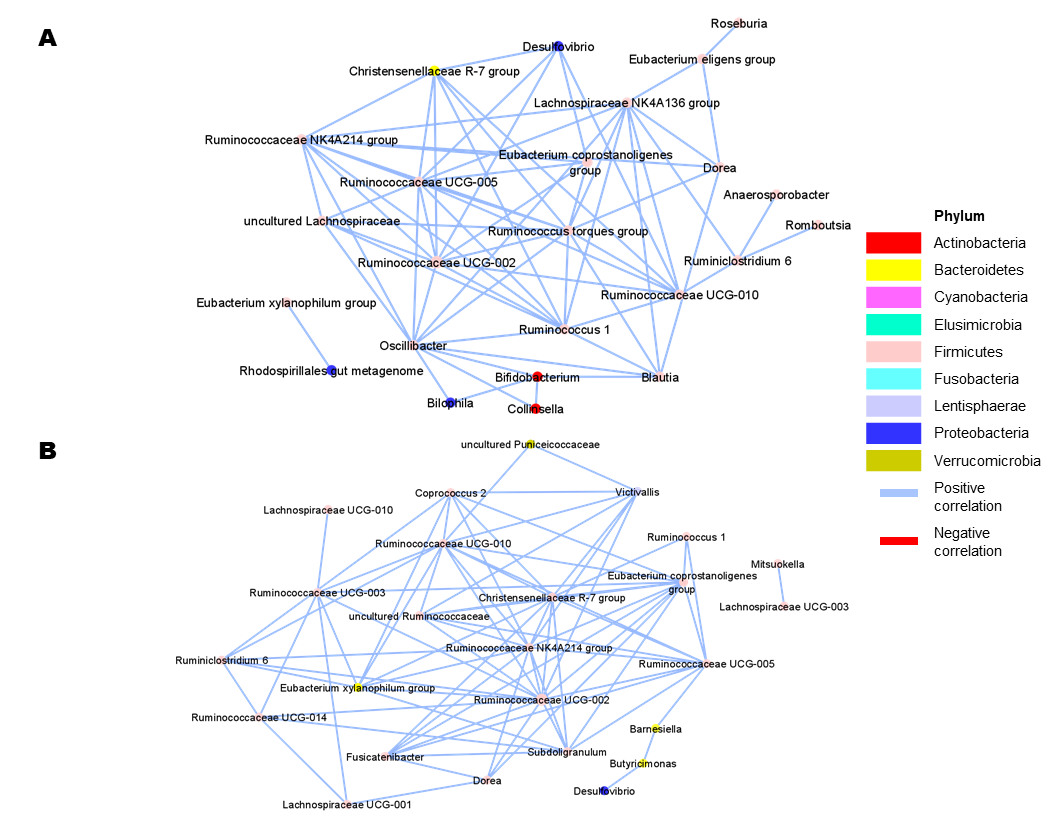

Supplement: Supplementary file 1 [file microorganisms-11-00136-s001.zip › Figure S4 - co-occurrence network of highly correlated clusters in both enterotypes.PNG]

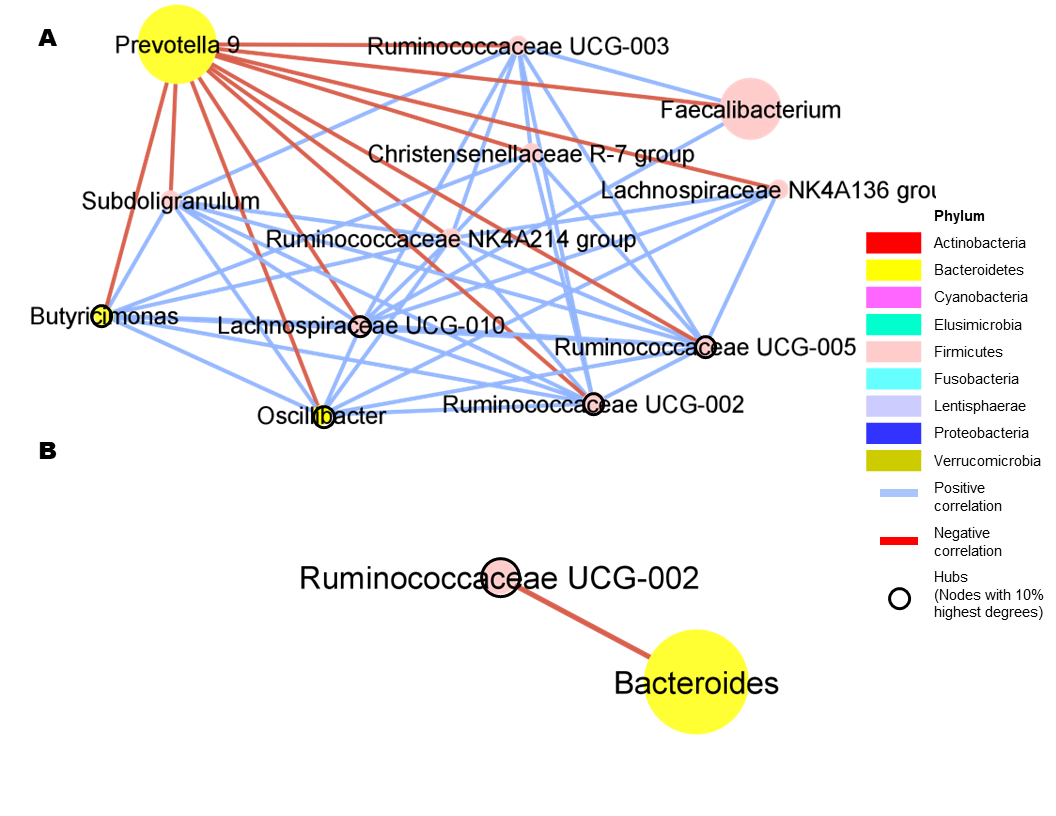

Supplement: Supplementary file 1 [file microorganisms-11-00136-s001.zip › Figure S5 - microbe¿Cmicrobe interactions of dominant taxa.PNG]
